# Features of Atrial Fibrillation Pathogenesis and Prognosis in Chronic Obstructive Pulmonary Patients during the COVID-19 Pandemic

**DOI:** 10.31083/j.rcm2402051

**Published:** 2023-02-06

**Authors:** Lusine G. Hazarapetyan, Parounak H. Zelveian, Svetlana V. Grigoryan

**Affiliations:** ^1^Department of Cardiology, Yerevan State Medical University named after Mkitar Heratsi, 0025 Yerevan, Armenia; ^2^Department of Arrhythmias, Scientific Research Institute of Cardiology named after Levon Hovhannisyan, 0014 Yerevan, Armenia

**Keywords:** COVID-19, chronic obstructive pulmonary disease, atrial fibrillation, risk factors, myocardial remodeling, systemic inflammation, fibrosis, hypoxia

## Abstract

**Background:**

Atrial fibrillation (AF) is observed in arterial hypertension, heart failure, ischemic heart disease, and pulmonary pathology, particularly, chronic obstructive pulmonary disease (COPD). COPD in turn is a risk factor for developing these cardiovascular diseases and various arrhythmias. In the coronavirus disease (COVID) situation, such comorbid patients are the most vulnerable group with a high risk of adverse outcomes. The relevance of the relationship between COPD and coronavirus infection is explained by the similarity of clinical and pathophysiological manifestations, creating more difficulties in diagnosing and determining rational treatment. The aim of the current study is to explore the role COPD plays in the onset and progression of AF, especially in the situation of COVID-19.

**Methods:**

We searched PubMed databases and included studies with information on comorbid patients suffering from COPD and AF, as well as similar patients in the context of COVID-19.

**Results:**

A modern view on the problem of comorbidity of COPD and AF is presented. In the presence of cardiorespiratory comorbidity, symptoms of mutual worsening of the clinical course are observed, due to the commonality of some links of pathogenesis, including hypoxia, hemodynamic disturbances, activation of the sympathoadrenal system, systemic inflammation, and development of fibrosis, leading to myocardial remodeling, a decrease in the effectiveness of the therapy, and a worsening prognosis, especially in the context of COVID-19.

**Conclusions:**

The results of a study of the features of the pathogenesis and course of AF in COPD are presented, as well as the formation and progression of this comorbid pathology in the context of the COVID-19 pandemic.

## 1. Introduction

Currently, the issues of comorbidity of pathological conditions, especially the combined lesions of the cardiovascular and bronchopulmonary systems, attract the attention of many clinicians. Non-valvular atrial fibrillation (AF) is observed in cardiovascular diseases (CVD) such as arterial hypertension (AH), ischemic heart disease (IHD), and heart failure (HF) and in pulmonary pathology, especially chronic obstructive pulmonary disease (COPD). Meanwhile, COPD in turn is a risk factor for developing hypertension, IHD, various arrhythmias, and HF. In the situation of the coronavirus infection pandemic, such comorbid patients are the most vulnerable group with a high risk of adverse outcomes. The combination of coronavirus infection with CVD and COPD creates more difficulties in diagnosing and determining rational treatment [1]. The situation is complicated by the lack of information and a considerable amount of often contradictory publications on these issues [2]. According to the European guidelines on coronavirus disease (COVID-19) [3], the pathogenesis of COVID-19 depends on generalized vasculitis of the microvasculature with the development of multiple thromboses and thromboembolism. Over the past 2 years, it has been revealed that almost half of COVID-19 patients had polymorbitism, which increased especially in severe cases [4, 5]. The relevance of the relationship between COPD and coronavirus infection COVID-19 is illustrated by the similarity of clinical pathophysiological manifestations, contributing to the complicated course of the disease [6, 7]. Clinicians have found that COVID-19 patients often have CVD and risk factors such as diabetes mellitus (DM) and obesity. According to a retrospective analysis of some Italian and Chinese studies, it was demonstrated that AH was observed in 16.9% of such patients, IHD in 30%, AF in 24.5%, stroke in 9.6%, and DM in 35.5% [8, 9]. Therefore, the problem of the relationship between these pathologies is very relevant; thus, the aim of the current review is to study the role of COPD in the onset and progression of AF, especially in the current situation of the COVID-19 pandemic.

## 2. Search for Publications and Selection of Studies

The information retrieval algorithm was developed according to the reporting requirements and regulations for systematic reviews and meta-analyses. We searched the PubMed database using the following keywords: “atrial fibrillation”, “chronic obstructive pulmonary disease”, and “COVID-19” for the period from January 1, 2020, to January 1, 2022. Based on the search results, 26 literature sources were analyzed: consensus papers, meta-analyses, literature reviews, articles, and clinical cases. Moreover, we analyzed studies in the database with information on comorbid patients with COPD and AF in the period from 2015 to 2022. Manual searches were also conducted among citations from individual review articles, meta-analyses, and consensus statements. 170 sources were analyzed. The inclusion criteria of studies in a systematic review followed by a meta-analysis were as follows: only randomized clinical trials; studies with access to full texts; with adult participants (18 years of age or older). A mandatory condition for the inclusion of publications in the meta-analysis was the provision of data on clinical outcomes, such as thromboembolic events and mortality. The minimum follow-up period in the studies was 6 months. Consequently, 50 sources were selected. Our review presents a modern approach to the problem of COPD and AF comorbidity and discusses the features and possible pathogenetic pathways contributing to the frequency of their development and mutual potentiation during the COVID-19 pandemic.

## 3. Results

### 3.1 AF Prevalence in COPD

AF is the most common type of cardiac arrhythmia, which prevails with age: it is 2–5% at the age of over 60 years and more than 17% at the age of 80–89 years [10, 11]. According to a previous study [12], there are 20.9 million men and 12.6 million women with AF (excluding asymptomatic patients). By 2030, there will be 14 to 17 million patients with AF in the European Union, with an annual increase of 120 to 215 thousand new patients. In the USA, there are about 5.2 million, and by 2030 their number will increase to 12.1 million [13]. The growing prevalence of AF has made it a worldwide problem. Such an increase in AF patients is most likely due to a significant improvement in diagnostic capabilities, an increase in life expectancy, and an increase in the number of HF patients. AF significantly worsens the quality and life expectancy of patients due to the possible development of thromboembolic complications.

Numerous authors [14, 15, 16] have indicated that risk factors for AF include older age, high blood pressure, and various comorbid conditions, especially concomitant heart and lung diseases. It was revealed that some electrophysiological, hemodynamic, and metabolic disorders resulting in electrophysiological and structural atrial remodeling can serve as pathogenetic mechanisms for the development and progression of AF [17, 18]. Thus, the progression of AF and its recurrence can increase morbidity and mortality from CVD [19]. Patients with progressive AF have been found to have more adverse cardiovascular outcomes and a high rate of hospitalization. The term “progression of AF” is understood as the process of development of paroxysmal AF in the direction of chronization of the process and its transition to a permanent form. The progression of AF depends on the process of atrial remodeling, which can be facilitated by hypertension, ischemia, and inflammation. Although AF often progresses from paroxysmal to persistent, more resistant forms, the rate and predictors of its progression in clinical practice are not well understood. The literature demonstrates that AF does not progress in only 5% of cases, relapses are observed in 40% of cases and become more frequent in 45% of cases, and finally, AF becomes permanent in 10% of cases [20]. The rate of AF progression depends on atrial remodeling, that is, the rate of progression of the underlying disease or the cause associated with the heart remodeling. The most informative scale for evaluating the progression and outcome of AF is the HATCH prognostic scale, in which the following endpoints are distinguished: AH: 1 point; age >75 years: 1 point; COPD: 1 point; HF: 2 points; transient ischemic attack: 2 points. The progression of AF is regarded as a poor prognostic sign. Meanwhile, mortality increases by 2 times, and hospitalization for cardiovascular episodes increases by 71% [21].

Some authors have noted that numerous traditional factors causing structural remodeling of the heart, such as age, AH, DM, HF, COPD, and high HATCH score correlate with a higher incidence and progression of AF. Therefore, comorbid patients are the most vulnerable group with a particularly high risk of adverse outcomes [22, 23]. The issue of the relationship of these pathologies is very relevant; thus, the present systematic review aims to explore the role of COPD in the occurrence and progression of AF, especially in the current pandemic situation. The authors have explored a systematic review and meta-analysis (420,000 patients) following international guidelines. All studies reporting the prevalence of COPD among AF patients were included. In 46 studies, pooled prevalence of COPD was 13% [95% confidence interval (CI) 10–16%, 95% prediction interval 2–47%]. COPD was associated with a higher prevalence of comorbidities and higher CHA2DS2-VASc score [odds ratio (OR) 0.77, 95% CI 0.61–0.98], as well as a higher risk of all-cause death [OR 2.22, 95% CI 1.93–2.55] [24].

It was found that the presence of CVD is not specifically associated with a high risk of coronavirus infection, but is associated with a higher risk of complications. In the European recommendations (2020) for COVID-19, it was demonstrated that non-specific, rapid heartbeat was a common manifestation of infection in 7.3% of patients with COVID-19, and cardiac arrhythmias occurred in 16.7%. Moreover, arrhythmias were more common in patients in intensive care units in comparison with other inpatients (44.4% versus 6.9%) [3, 25]. One of the comorbid conditions in patients with AF is COPD, which belongs to a group of diseases causing airflow limitation and thereby contributing to respiratory failure. These are mainly chronic bronchitis and emphysema. COPD spreads widely among AF patients (about 25%), has common risk factors, and increases overall morbidity and mortality in this population [26]. COPD is characterized by a progressive course, which is associated with increased chronic inflammation in the airways and lungs. COPD takes the third place in the structure of mortality worldwide, while 30% of deaths in COPD are due to cardiovascular pathology [27, 28]. Hypoxia, oxidative stress, and inflammation are major factors in the pathophysiology of COPD, and these processes also play a vital role in atrial remodeling, that is, being actively involved in the onset and progression of AF. According to the WHO, more than 3,000,000 people died from COPD, which is about 5% of all deaths [28]. The EUR Observational Research Program investigated the prevalence of COPD in patients participating in the pilot study of the AF registry in this study [29]. After 1 year of follow-up, it was observed that, in the total cohort, the diagnosis of COPD was registered in 339 (11.0%) patients with AF. Patients with AF and COPD were more susceptible to risk factors and comorbidities, including DM (*p *< 0.0001) and HF (*p *< 0.0001). At follow-up, patients with AF and COPD had a higher risk of CVD and all-cause death (both *p* values < 0.0001).

### 3.2 Predictors of AF in Comorbidity with COPD

COPD severity was defined according to the GOLD classification, and arrhythmias were classified according to the current ESC clinical guidelines. Of the 7441 patients (aged 64 ± 16 years) included in the study, COPD was diagnosed in 3121 patients (41.9%). The presence and severity of COPD were associated with an increased likelihood of AF/atrial flutter in comparison with patients without COPD (23.3% versus 11.0%, p < 0.0001) and with non-sustained ventricular tachycardia (13.0% versus 5.9%, *p *< 0.0001) [30]. Several epidemiological studies have been carried out for analyzing the relationship between AF and impaired lung function. In earlier PAISLEY studies (15406 COPD patients), it was found that AF patients had a lower forced expiratory volume in one second (FEV1) [31]. The COPENHAGEN study showed an obvious relationship between the severity of bronchial obstruction and the occurrence of AF, which was observed 2 times more often in patients with FEV1 less than 60% [32].

In the TAKAHATA study, FEV1 was found to be an independent risk factor for AF, and its prevalence was higher in the airflow limitation group than in patients with left ventricular (LV) hypertrophy (14.3% versus 4.4%) [33, 34]. It was demonstrated that the frequency of occurrence of AF is inversely proportional to FEV1.

COPD is not only an independent predictor of major adverse cardiovascular events, but also a predictor of AF frequency. Thus, the prevalence of AF was higher in patients with severe airway obstruction in comparison with mild or moderate ones. Moreover, in COPD, supraventricular arrhythmias are observed in 77%, AF was observed in 8% of patients, and the risk of AF hospitalization was higher among patients with a low FEV1 <60% [35]. Some researchers [36] presented a review of 60 literature sources regarding the comorbidity of COVID-19 and COPD. It is evident that COPD patients have increased the expression of the angiotensin-converting enzyme 2 receptor in the lungs, which may contribute to a greater susceptibility to COVID-19. In COPD patients, endothelial cell dysfunction and a tendency to thrombosis have been identified, which may also be a risk of adverse COVID-19 outcomes.

### 3.3 Inflammation and Fibrosis

It is known that COPD patients have considerable prerequisites for myocardial damage, including hypoxia and oxidative stress, toxic effects of bronchopulmonary inflammation, hypercatecholaminemia due to activation of sympathoadrenal system (SAS), endothelial dysfunction, hemodynamic disturbances, and pulmonary hypertension [37].

Furthermore, this combination of COPD and CVD is characterized by the commonality risk factors including smoking and a reduction in FEV1, which is regarded as a predictor of the development of CVD [34, 38]. Numerous researchers noted that mortality in COPD patients is mainly caused by CVD, not respiratory failure, and that HF and acute coronary insufficiency are more likely to develop. Moreover, COPD is the main contributor to the increase in overall mortality from AF [38, 39]. The authors presented that the relative risk of mortality increases when COPD is combined with paroxysmal AF, especially against the background of pulmonary hypertension, since oxidative stress and chronic systemic inflammation play a vital role in the pathogenesis of both AF and COPD, contributing to the destruction of atrial myocytes and fibrosis and, consequently, the progression of both pathological processes.

Nowadays, the relationship between endothelial dysfunction and systemic inflammation has been proven. Thus, in COPD patients, one of the key factors that damage the vascular wall is toxic components that cause oxidative stress, resulting in endothelial dysfunction. Meanwhile, the activity of nitric oxide synthase is suppressed, leading to a reduction in the synthesis of nitric oxide, as an active vasodilator, and suppressing the expression of cell adhesion, thereby providing an antiproliferative and antithrombotic effect [40]. Moreover, due to hypoxia, SAS is activated, causing vasoconstriction of blood vessels. This effect, in turn, potentiates the effect on the vascular wall of other endogenous mediators, including histamine, serotonin, angiotensin II, and catecholamines, which are produced against the background of hypoxia. Hypoxia also decreases renal blood flow and activates the renin-angiotensin-aldosterone system (RAAS), contributing to oxidative stress and vasoconstriction [41]. The authors also identified an increase in the level of cortisol and aldosterone in COPD patients. The main factor triggering the process of lipid peroxidation is the SAS, which is activated in response to hypoxia, promoting lipid peroxidation of cell membranes and their damage. This fact results in producing proinflammatory cytokines including interleukin-1 (IL-1), interleukin-6 (IL-6), and tumor necrosis factor-alpha (TNF-α), thereby increasing the expression of cell adhesion and activation of smooth muscle cell proliferation. The role of endothelial dysfunction in developing cardiovascular pathology in COPD patients was studied. A relationship was found between the concentration of the marker of endothelial dysfunction endothelin-1 and the presence of coronary artery disease in COPD patients. The relationship between the concentration of the vascular type-1 cell adhesion molecule and the occurrence of paroxysmal AF in patients after coronary artery bypass grafting was determined [42, 43, 44]. Therefore, it can be considered that a possible single pathogenetic mechanism in patients with AF and COPD is systemic inflammation, which links the severity of broncho-obstructive syndrome and the atherogenetic process, increasing the risk of developing various arrhythmias.

Numerous authors in their studies have illustrated that the levels of IL-6 and high sensitive C-reactive protein (hsCRP) contribute to the development of AF, as well as the risk of its recurrence. It is found that patients with positive hsCRP (>0.6 mg/dL) were 5.91 times more likely to develop AF compared to those with negative hs-CRP [42]. The authors believed that hsCRP levels can help predict the risk of AF recurrence. In the study, the authors also pointed out that cellular inflammatory infiltration of the atrial myocardium in patients with AF remarkably surpasses that in individuals without AF (*p *< 0.01). The revealed cytological picture of the myocardium is characterized by the replacement of cardiomyocytes with fibrocytes in the atria. It turns out that the accumulation of glycogen and atrial fibrosis are proven risk factors for the progression of AF [44]. Regarding the COVID-19 pandemic, utilizing anti-fibrotic drugs, such as bromhexine and spironolactone, is proposed, which may contribute to the possible prevention of pulmonary fibrosis. However, it is believed that so far there is no evidence base on how drugs utilized for treating COPD affect the onset and progression of AF. The European guidelines (ESC 2020) [3] indicated that CVD damage was diagnosed in 40% of patients who died from COVID-19 infection. Moreover, it was noted that although the causes of developing arrhythmias in COVID-19 are not well defined, they can be due to metabolic disorders, hypoxia, and neurohormonal or inflammatory changes in cases of a viral infection in patients with CVD and without their history. The European guidelines [3] in the section “Worsening of the course of chronic CVD in respiratory viral infections” draw attention to the high risk of complications due to atherosclerotic plaque rupture in virus-induced inflammation in patients with HF and CAD. Therefore, drugs that stabilize atherosclerotic plaques, such as statins, are recommended. The risk of thrombotic complications (e.g., stent thrombosis) due to the pro-coagulant effect of inflammation is indicated. Furthermore, the role of microvascular damage due to hypoperfusion, increased vascular permeability, angiospasm, and the direct damaging effect of the virus on the coronary artery endothelium has been revealed. Meanwhile, utilizing antiplatelet and anticoagulant therapy can help reduce this risk. In the section “Relationships of COVID-19 with cardiovascular disease” of these recommendations, special attention is paid to the occurrence of malignant arrhythmias in patients with elevated troponin, which should raise the suspicion that the patient has myocarditis.

### 3.4 Metabolic and Electrolyte Disorders 

Studies have demonstrated that as hypoxia increases in COPD patients, the expression of matrix metalloproteinases increases, which are involved in the regulation of atrial structural remodeling. Hypoxia can also cause narrowing of the pulmonary arteriolar process, gradually resulting in pulmonary hypertension. It turned out that higher values of partial pressure of carbon dioxide and systolic pressure in the pulmonary artery significantly increase the possibility of AF [42]. The authors believed that suboptimal lung function, hypercapnia, and high values of systolic pressure in the pulmonary artery may be independent predictors of AF [38, 43, 44]. Of particular interest are the results of studying the concentration of potassium and magnesium in the blood in COPD patients. It has been proven that a reduction in potassium concentration is an extremely common electrolyte imbalance in COPD patients. The level of potassium in the blood serum also affects the potential of the cell membrane and contributes to the appearance of various arrhythmias, including AF. At the same time, a low level of potassium in the blood serum increases the duration of the P wave, which can also be a predictor of the incidence of AF [45]. P wave dispersion, which is the difference in maximum and minimum P wavelength, is also an independent risk factor attributed to the development of AF. It can be assumed that the dispersion of the P wave may be a marker of prognosis, prevention, and rational treatment of exacerbations of COPD in comorbid patients with cardiac pathology.

### 3.5 Pulmonary Hypertension

It is established that COPD patients more often develop pulmonary hypertension, which is based on hypoxia, polycythemia, and endothelial dysfunction. Thus, as a result of hypoxia, vasoconstriction is observed, and the proliferation of vascular smooth muscle cells is activated, leading to remodeling of the pulmonary vascular bed and destruction of the lung parenchyma. Polycythemia, contributing to an increase in blood viscosity, increases pulmonary resistance. It is the risk factor for developing thromboembolism, against which secondary pulmonary hypertension may occur [46]. In addition, pulmonary hypertension may worsen the condition in patients with AF and COPD by increasing right atrial overload or stretch in COPD patients [47]. Accordingly, overload and dilatation of the left atrium (LA) are observed, and the time of conduction of electrical impulses through the atria increases, which may be the cause of AF. The number of AF recurrences is associated with the severity of COPD, regardless of effective treatments. In a previous study [48], the association between respiratory failure, LV dysfunction, and arrhythmias has been examined in COPD patients. It was demonstrated that LV diastolic dysfunction correlates with the severity of bronchial obstruction. In previous work [49], a correlation was also observed between the worsening of LV diastolic dysfunction and the progression of the course of AF. Therefore, dilatation of both the right and left ventricles in COPD patients may have prognostic significance in terms of the likely development of AF in these patients. Therefore, it was indicated that the frequency of AF correlated with FEV1 (R = –0.348; *p* = 0.013), reduced blood oxygen saturation (R = –0.356; *p* = 0.011), hs-CRP level (R = 0.442; *p* = 0.001), the size of both atria (*p *< 0.001), LV isovolumetric relaxation time (R = 0.350; *p* = 0.022), right ventricular size (R = 0.478; *p *< 0.001) and systolic pressure in the pulmonary artery (*p *< 0.001) [50]. Earlier studies [34, 35, 38, 39] revealed that FEV1 has a statistically significant effect on the LA volume index (*p* = 0.008) and LV isovolumetric relaxation time (*p* = 0.005). The authors believed that severe bronchial obstruction, hypoxemia, systemic inflammation, vascular l, and myocardial remodeling are risk factors contributing to the onset and progression of AF in COPD patients.

The European guidelines [3] identified possible causes of cardiac arrhythmias in COVID-19 as follows:

• angiotensin-converting enzyme 2 signaling pathways involved in the heart damage cascade (reduction in the expression and dysregulation of RAAS);

• pathological systemic inflammatory response, manifested by a “cytokine storm” caused by an imbalance in the response of type 1 and type 2 T-helper cells and leading to multiple organ failure, including damage to the cardiovascular system;

• respiratory dysfunction and hypoxia (oxidative stress, intracellular acidosis, and damage to mitochondria), contributing to damage to cardiomyocytes;

• imbalance between increased metabolic demands and decreased cardiac reserve.

Perhaps further evidence-based studies will increase the list of these mechanisms.

## 4. Conclusions

The current review presents the results of studies for identifying the changes in the clinical picture with the mutual influence of AF and COPD, as well as the factors in the formation and progression of comorbid pathology, especially in the context of the COVID-19 pandemic. It should be noted that sufficient knowledge of the specific mechanisms of AF in patients with COPD will assist in providing rational orientation of clinicians to specific pathophysiological links. The results of studies on the identification of changes in the clinical picture with the mutual influence of AF and COPD and factors of the formation and progression of comorbidity were analyzed. With cardiorespiratory comorbidity resulting in symptoms of mutual aggravation, features of the clinical course are formed due to the commonality of some links of pathogenesis, including hypoxia and oxidative stress, hemodynamic disturbances, SAS activation, systemic inflammation, fibrosis, and, ultimately, myocardial remodeling, leading to a reduction in efficiency of therapy and worsening disease prognosis. All of the above factors and possible mechanisms indicate the complexity of the processes of AF arrhythmogenesis in COPD patients, as well as the possibility of preventing AF in such patients. Few studies have shown that viral infection contributes to the destabilization of existing cardiovascular diseases and COPD, that is, those comorbid conditions which are significant prerequisites for heart damage. Prevention of these conditions is a rational direction. However, its possibilities are still to be explored. It is hoped that the use of drugs that can prevent or reduce structural remodeling and atrial fibrosis will allow cardiologists to move closer to therapeutic ideals in managing AF in COPD patients, especially in the context of the COVID-19 pandemic.

## Abbreviations

AF, atrial fibrillation; AH, arterial hypertension; CI, confidence interval; COPD, chronic obstructive pulmonary disease; COVID-19, coronavirus disease 2019; CVD, cardiovascular diseases; DM, diabetes mellitus; FEV1, forced expiratory volume in one second; HF, heart failure; hsCRP, high sensitive C-reactive protein; IHD, ischemic heart disease; IL-1, interleukin-1; IL-6, interleukin-6; LA, left atrium; LV, left ventricle; OR, odds ratio; RAAS, renin-angiotensin-aldosterone system; SAS, sympathoadrenal system; TNF-α, tumor necrosis factor-alpha.
